# Tuberous sclerosis diagnosed by incidental computed tomography findings of multifocal micronodular pneumocyte hyperplasia: a case report

**DOI:** 10.1186/1752-1947-6-352

**Published:** 2012-10-16

**Authors:** Makoto Ishii, Koichiro Asano, Nobufumi Kamiishi, Yuichiro Hayashi, Daisuke Arai, Mizuha Haraguchi, Hiroaki Sugiura, Katsuhiko Naoki, Sadatomo Tasaka, Kenzo Soejima, Koichi Sayama, Tomoko Betsuyaku

**Affiliations:** 1 Division of Pulmonary Medicine, Department of Medicine, Keio University School of Medicine, 35 Shinanomachi, Shinjuku-ku, Tokyo, 160-8582, Japan; 2 Department of Pathology, Keio University School of Medicine, 35 Shinanomachi, Shinjuku-ku, Tokyo, 160-8582, Japan; 3 Department of Diagnostic Radiology, Keio University School of Medicine, 35 Shinanomachi, Shinjuku-ku, Tokyo, 160-8582, Japan

**Keywords:** Computed tomography (CT), Multifocal micronodular pneumocyte hyperplasia (MMPH), Tuberous sclerosis.

## Abstract

**Introduction:**

The majority of multifocal micronodular pneumocyte hyperplasia associated with tuberous sclerosis complex is diagnosed with the classical clinical triad of seizures, mental retardation, and skin lesions. We report a rare case of tuberous sclerosis complex with no classical clinical findings, which was diagnosed through incidental computed tomography findings of multiple nodular lesions of multifocal micronodular pneumocyte hyperplasia.

**Case presentation:**

A chest computed tomography scan of a 51-year-old Japanese woman showed multiple nodular ground-glass opacities that were not seen on chest X-ray. Video-assisted thoracoscopic surgery was performed. A histological examination demonstrated type II pneumocyte hyperplasia with thickened fibrotic alveolar septa, which was consistent with multifocal micronodular pneumocyte hyperplasia. Brain magnetic resonance imaging displayed multiple cortical tubers, and abdominal computed tomography showed bilateral renal angiomyolipoma. Our patient was finally diagnosed as having tuberous sclerosis complex with multifocal micronodular pneumocyte hyperplasia, although she had no episodes of epilepsy, no skin lesions, and no family history.

**Conclusions:**

Multifocal micronodular pneumocyte hyperplasia with latent tuberous sclerosis complex should be considered in the differential diagnosis of multiple ground-glass opacities.

## Introduction

Tuberous sclerosis complex (TSC) is an autosomal-dominant disease, first described by Bourneville in 1880 [1], characterized by hamartomatous lesions in various organs such as the skin, retina, kidney, central nervous system, heart, and lungs [2]. The major pulmonary manifestations of TSC are lymphangioleiomyomatosis (LAM) and, to a lesser extent, multifocal micronodular pneumocyte hyperplasia (MMPH) [3,4]. These pulmonary manifestations are reported to occur in 2.3 percent of cases [3]. However, studies of high-resolution computed tomography (CT) screening and autopsy in TSC cases have suggested that the incidences of MMPH and LAM associated with TSC are underestimated [4]. MMPH is a benign hamartomatous lung disease usually found in younger or middle-aged women associated with TSC [5]. The typical CT findings are multiple nodular ground-glass opacities (GGOs). MMPH is generally diagnosed as a pulmonary manifestation of TSC, with typical clinical findings in the central nervous system or skin, such as mental retardation, seizures, and/or facial angiofibroma, although few such patients have any clinical symptoms related to MMPH itself [6]. Here, a case of TSC without the classic clinical triad (mental retardation, seizures, and facial angiofibroma), which was diagnosed as TSC with MMPH through incidental CT findings of multiple GGOs, is reported.

## Case presentation

**Figure 1:**
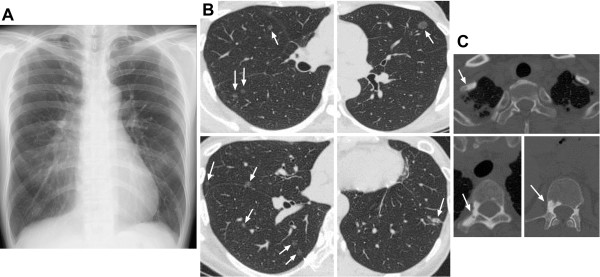
**Chest radiograph and computed tomography findings at our patient’s first visit. ** (**A**) Chest radiograph demonstrating pleural wall thickening on the right side of the apex and upper lung field. (**B**) Multiple nodular lesions, typically 10mm or less in diameter, can be observed throughout the lung fields on high-resolution chest computed tomography scan (arrows). The nodular lesions are mostly ground-glass opacities, while some have a higher density. (**C**) A chest computed tomography scan demonstrating the bone sclerosis lesions at Th3, Th6, and the first costal bone (arrows).

A 51-year-old Japanese woman presented to our facility for her regular physical examination. Chest radiographs showed pleural wall thickening on the right side of the apex and upper lung field, which had been seen for several years on her annual regular examinations (Figure 1A). She had had a mild dry cough for two weeks without symptoms of a common cold such as sore throat and fever. She was a smoker (20 cigarettes per day for 20 years). She had no history of lung disease, such as bacterial pneumonia or pulmonary tuberculosis.

A chest high-resolution CT scan was performed for detailed examination and showed multiple nodular lesions (mostly GGOs) throughout the lung fields, approximately 1cm or less in diameter, which were not detected on chest radiographs (Figure 1B). No cystic lesions, pleural effusions, or mediastinal lymph node swellings were observed. Bone sclerosis lesions were detected at Th3, Th6, and the first costal bone (Figure 1C). The findings seen on the right side of the upper lung fields on chest radiographs were evaluated as old inflammatory changes based on the chest CT scan; their etiology seemed to be different from the multiple nodular GGOs (data not shown).

Our patient was referred to our hospital for further investigation and treatment. The differential diagnoses of the lung GGOs were atypical adenomatous hyperplasia (AAH), highly differentiated adenocarcinoma *in situ* (AIS), lymphoproliferative disease, and MMPH. Her physical findings were normal. Breath sounds were normal, and no rales were heard on chest auscultation. No skin lesions such as facial angiofibroma and hypomelanotic macules were observed. A neurological examination showed no abnormalities. She had no intellectual disability and no history of epilepsy or other diseases. There was no family history of TSC. There were no abnormal laboratory test results at the time of her first visit to our hospital. Arterial blood gas analysis results were also normal. Pulmonary function tests were normal except for a mild decrease in diffusing capacity for carbon monoxide (DLCO).

**Figure 2:**
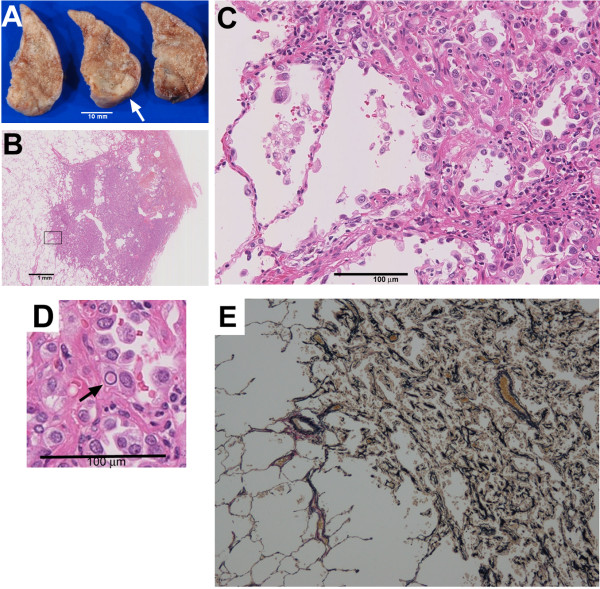
**Histopathological findings from video-assisted thoracoscopic surgery specimens of the left lung S9. ** (**A**) Macroscopic examination showing white-tinged lesions (arrow). (**B-D**) Microscopic histological examination demonstrating that the lung lesion is well demarcated (**B**) and consists of papillary growth of hyperplastic type II pneumocytes (**C**) with nuclear inclusion bodies (arrow in (**D**). Fibrous thickening of alveolar septa accompanied by increased elastic fibers can also be observed, leading to collapse of the alveolar space (**C**, **D**). (**E**) Elastica van Gieson staining confirming that elastic tissue fibers are evident.

To further investigate the multiple nodular lung lesions, video-assisted thoracoscopic biopsies of the left upper lobe (S5) and the left lower lobe (S9) were performed. The lesions were found to be tinged white on macroscopic examination (Figure 2A). Microscopically, the nodules were well demarcated (Figure 2B) and consisted of papillary growths of hyperplastic type II pneumocytes accompanied with nuclear inclusion bodies and fibrous thickening of alveolar septa accompanied with increased elastic fibers, resulting in the collapse of the alveolar space (Figure 2C,D). Elastica van Gieson staining revealed increased elastic tissue fibers in the lesions (Figure 2E). No cystic lesions were detected. Human Melanoma Black (HMB)-45-positive cells, a characteristic feature of LAM, were not observed on immunohistochemical staining (data not shown). These histological findings were consistent with MMPH.

**Figure 3:**
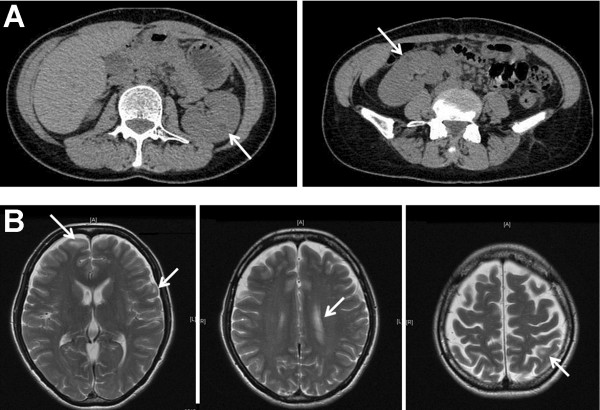
**Evidence for kidney and brain involvement on computed tomography and magnetic resonance imaging. ** (**A**) A non-enhanced abdominal computed tomography scan showing bilateral low-density areas with negative Hounsfield unit values inside the kidney, suggesting a renal angiomyolipoma (arrows). (**B**) Brain magnetic resonance imaging demonstrating multiple small, nodular, T2 high-intensity areas in cortical, subcortical, and subependymal zones, suggesting cortical and subcortical tubers (arrows).

A subsequent CT scan suggested that there were bilateral renal angiomyolipomas (Figure 3A). Brain magnetic resonance imaging (MRI) demonstrated multiple high intensity areas of cortical and subcortical tubers (Figure 3B). All of these findings, including bone, kidney, and brain manifestations, are typical features of TSC [7], and the latter two findings are major diagnostic criteria for TSC [8].

Finally, from the comprehensive assessment including the histological findings of lung nodular lesions and the CT and MRI findings of TSC, such as renal angiomyolipoma, cortical and subcortical tubers, and vertebral bone sclerosis, our patient was diagnosed as having TSC with MMPH, though she had no typical classical manifestations of TSC such as seizures, mental retardation, skin lesions, or a family history of TSC. Our patient is now under routine follow-up with no medication, and is asymptomatic.

## Discussion

Here, we describe a rare case of TSC with no classical clinical findings of mental retardation, seizures, and facial angiofibroma, which was diagnosed through incidental CT findings of multiple nodular lesions of MMPH. TSC is an autosomal dominant disorder characterized by multiple hamartoma lesions distributed throughout the body, especially the skin, retina, kidney, central nervous system, heart, and lungs [1]. Our patient met two of the major diagnostic criteria (renal angiomyolipoma and cortical tubers) for TSC and was finally diagnosed as having TSC with MMPH in this case.

The classical clinical triad of TSC symptoms are mental retardation, seizures, and cutaneous angiofibroma (formerly called adenoma sebaceum), reported by Vogt in [9]. Mental retardation and seizures are both neurologic manifestations of TSC [10]. The overall incidence of mental retardation is 38 percent to 80 percent in TSC [10], while epilepsy is one of the most prevalent manifestations of TSC, occurring in more than 80 percent to 90 percent of patients with TSC [2,11]. These neurological manifestations are highly related to cortical tubers, which are detected in 80 percent of patients [2]. In this case, neither mental retardation nor seizures were detected, although cortical and subcortical tubers were found on brain MRI scans. The skin lesions of TSC, such as facial angiofibroma and hypomelanotic macules, are detected in more than 90 percent of patients with TSC [2], but they were not observed in our patient’s case. It has been reported that 6 percent of patients with TSC have none of these three findings [12]. These previous reports confirmed that the majority of patients with TSC are generally suspected and diagnosed based on these clinical findings. However, TSC may be underdiagnosed in patients without any of these symptoms even when MMPH coexists, because MMPH is generally asymptomatic, and the lesions are often difficult to see on chest radiographs [6].

TSC is an autosomal dominant disease that is associated with gene mutations of *TSC1* or *TSC2*, encoding hamartin and tuberin, respectively [2]. However, two-thirds of cases are caused by sporadic mutations [13], and this may also contribute to the underdiagnosis of TSC cases without the classic triad. In our patient’s case there was no family history of TSC, suggesting that this case was sporadic, though TSC gene mutations were not evaluated in our patient.

There are two major lung manifestations of TSC: LAM and MMPH [4]. LAM is a rare lung disease characterized by diffuse proliferation of abnormal smooth muscle-like cells and cystic destruction of the lung [14]. LAM occurs sporadically or as a pulmonary manifestation of TSC; it is usually diagnosed in early adult women and initially manifests as dyspnea and pneumothorax [2]. Both LAM and MMPH often co-exist [4], but this was not the case in our patient.

MMPH is another pulmonary manifestation of TSC characterized by multiple nodular GGOs on chest radiographs or CT scans because of proliferation of type II pneumocytes and fibrous thickening of alveolar septa. It is often difficult to distinguish MMPH from AAH or AIS histologically [15]. However, the finding of increased elastic fibers in alveolar septa, which contribute to the collapse of the alveolar space, is more common in MMPH than in AAH or AIS [15]. In fact, an increase of elastic fibers in the alveolar septa seemed evident in our patient’s case. Since the lung shadows of MMPH remain unchanged or slowly progressive after long-term follow-up, and the prognosis of MMPH is relatively good [15], the clinical diagnosis of latent TSC is critical for distinguishing MMPH from AAH or AIS.

## Conclusions

We report a rare case of MMPH associated with TSC without the classical clinical triad of seizures, mental retardation and skin lesions. Since MMPH is generally asymptomatic, and it is often difficult to detect multiple GGOs of MMPH lesions on chest X-rays, and since MMPH is generally suspected when the patient is diagnosed as having TSC with the classical triad, it appears that the incidence of TSC may be underestimated without such symptoms. TSC should be suspected when the patient has multiple nodular GGOs. MMPH should be carefully distinguished from AAH or AIS even when the patient has no history of epilepsy or skin lesions or a family history.

## Consent

Written informed consent was obtained from the patient for publication of this case report and any accompanying images. A copy of the written consent is available for review by the Editor-in-Chief of this journal.

## Abbreviations

TSC: Tuberous sclerosis complex; LAM: Lymphangioleiomyomatosis; MMPH: Multifocal micronodular pneumocyte hyperplasia; CT: Computed tomography; GGOs: Ground-glass opacities; AAH: Atypical adenomatous hyperplasia; AIS: Adenocarcinoma in situ; DLCO: Diffusing capacity for carbon monoxide.

## Competing interests

All authors declare that they have no competing interests.

## Authors’ contributions

MI, KA, YH, KN, ST, and KS participated in the clinical diagnosis. YH performed the histological examination. MI and KA participated in patient care and follow-up. MI, KA, and TB contributed to drafting of the manuscript. All authors read and approved the final manuscript.
